# Incorporation of Caprylic Acid into a Single Cell Oil Rich in Docosahexaenoic Acid for the Production of Specialty Lipids

**DOI:** 10.17113/ftb.58.04.20.6546

**Published:** 2020-12

**Authors:** Daniela Kanno Mathias, Jacqueline Piazentin Costa, Carolina Rodrigues Calvo, Roberta Claro da Silva, Attilio Converti, Nadia Segura, Iván Jachmanián, Luiz Antonio Gioielli, Juliana Neves Rodrigues Ract

**Affiliations:** 1 Department of Biochemical and Pharmaceutical Technology, School of Pharmaceutical Sciences, University of São Paulo, Av. Prof. Lineu Prestes, 580, 05508-000 São Paulo, Brazil; 2 Department of Family and Consumer Sciences, North Carolina Agricultural and Technical State University, 1601 E. Market Street, NC 27411 Greensboro, USA; 3 Department of Civil, Chemical and Environmental Engineering, Pole of Chemical Engineering, Via Opera Pia 15, 16145 Genoa, Italy; 4 Department of Food Science and Technology, School of Chemistry, University of the Republic (UDELAR), Av. Gral Flores 2124, Casilla de Correos 1157, 11800 Montevideo, Uruguay

**Keywords:** single cell oil, new source of docosahexaenoic acid, structured lipids, acidolysis, caprylic acid

## Abstract

**Research background:**

New sources of docosahexaenoic acid have recently been investigated aiming at infant formula fortification and dietary supplementation, among which the single cell oil with 40-50% of this acid.

**Experimental approach:**

For this purpose, such an oil was blended with caprylic acid in amount substance ratio ranging from 1:1 to 5:1 and the blends were interesterified using either Novozym 435 or Lipozyme TL IM as the catalyst. The influence of the amount of excess free caprylic acid in the substrate, as well as the type of enzyme on the triacylglycerol rearrangement resulting from the synthesis of the structured lipids were evaluated.

**Results and conclusions:**

The regiospecific lipase Lipozyme TL IM seemed to induce transesterification among single cell oil triacylglycerols preferably by acidolysis with caprylic acid, which was directly proportional to the ratio of this acid in the substrate. In reactions catalyzed by the non-regiospecific lipase Novozym 435, a higher incorporation of caprylic acid into single cell oil triacylglycerols was observed than when using Lipozyme TL IM, independently of the oil/caprylic acid molar ratio.

**Novelty and scientific contribution:**

These results revealed the importance of combining the choice of the type of lipase, either regiospecific or not, with the amount ratios of free fatty acids and the substrate in acidolysis when aiming to produce structured lipids as a source of docosahexaenoic acid.

## INTRODUCTION

Docosahexaenoic acid (DHA), a long-chain polyunsaturated fatty acid (PUFA) of the omega-3 family with shortened name C22:6 *n*-3, is a major structural component of neural membranes and retina and a particularly important nutrient in terms of biological functions during infant development (*1*). In the 1980s, the major source of this fatty acid (FA) in the human diet was from fish or fish oil capsules, the latter having poor acceptance on the market due to their strong organoleptic characteristics. Attempts to apply fish oil as a food ingredient were also largely unsuccessful, owing to their unpleasant fishy taste and odor as well as to emerging concerns with the levels of environmental contaminants (polychlorinated biphenyls (PCBs), dioxins, and mercury) reported in fish (*2*).

Therefore, alternative PUFA-rich lipids used as food additives to improve the FA composition of foods have recently been investigated, aiming at infant formula fortification and dietary supplementation. Food fortification with PUFAs can be reached in different ways such as the extraction and addition of PUFA-rich microbial lipids. As human population is constantly growing and the PUFA sources are limited, microbial oils turned out to be a safe and well-defined choice in the food industry as a PUFA source, also due to their sustainable production independent of climatic or seasonal changes (*3*).

Among the commercially available sources of PUFAs, the DHA from a single cell oil (DHASCO), derived from the microalgae *Crypthecodinium cohnii*, contains 40-50% DHA (*4*, *5*). Studies performed in rats by Arterburn *et al*. (*6*-*8*) provide evidence that DHASCO is a safe source of dietary DHA, as it did not appear to have any mutagenic, genotoxic or teratogenic potential even at doses 100 times higher than the expected uptake levels. In addition, DHASCO DHA was found to be bioavailable, resulting in a significant increase of its levels in liver, heart and brain after 90 days of administration in rats.

Single cell oils (SCO) are defined as edible oils obtainable from microorganisms that can accumulate lipids to more than 20% (by mass) in their dry cell mass. These oils are similar in composition to the oils and fats of animal or vegetable origin or may contain proportions of long chain polyunsaturated FAs even larger than those found in such oils (*9*). Some specific microorganisms, including bacteria, microalgae, yeasts and filamentous fungi are capable of growing and producing SCO from agro-industrial wastes and byproducts, hence combining valorization of these residues, benefits for the environment, and production of potentially high value-added lipids (*10*). Currently, SCOs are important nutraceutical ingredients of non-animal origin in infant formulas as sources of arachidonic acid (20:4 *n*-6, ARA) and DHA (*11*).

Human milk supplies DHA for newborns, but many commercially available infant formulas do not contain enough of this FA in their formulation. In studies performed by Carlson *et al*. (*12*-*14*), supplementation of infant formula with marine oil containing DHA and eicosapentaenoic acid (20:5 *n*-3, EPA) was successful in improving the DHA status and visual function, but affected the ARA status and growth, which could result in subsequent inhibition of events that are crucial to normal development. Despite its beneficial effects on the human health (*15*), EPA was shown to metabolically interfere with the efficacy of DHA uptake (*16*), therefore the marine oil that contains EPA and DHA in similar mass fractions may not be completely satisfactory sources of DHA for therapies based on this FA. Thus, one of the challenges in this field is to search for SCOs containing DHA as the main FA in their composition (*17*).

An interesting approach to structured lipid development in the functional food industry lies in the insertion of medium-chain fatty acids (MCFAs, C6-C12) into the *sn*-1,3 positions of triacylglycerols (TAGs) of the oil containing high amounts of unsaturated or polyunsaturated long-chain fatty acids (LCFAs, C14-C24), while original FAs are ideally kept in the *sn*-2 position (*18*). TAGs resulting from these reactions are reported to effectively release the desired FAs and can thus be used in malabsorption syndromes, providing energy as well as essential FAs in a more absorbable manner (*19*). In addition, free FAs (FFAs) released from dietary food during absorption are metabolized more easily if they possess medium- or short-chains, while LCFA monoacylglycerols can be absorbed directly. Therefore, essential or desired FAs are most efficiently utilized from the *sn-*2 position in TAGs (*20*). Acidolysis reactions catalyzed by *sn-*1,3 regiospecific lipases are one of the most commonly used methods to achieve this goal (*21*).

More recently, structured TAGs enzymatically enriched with EPA and DHA have been produced using multistage reaction routes. Castejón and Señoráns (*22*) used camelina oil to enzymatically produce 2-monoacylglycerols with high α-linolenic acid content using Lipozyme TL IM, which then reacted with EPA and DHA ethyl esters aiming to produce very long-chain structured lipids as functional ingredients. Wang *et al.* (*23*) first obtained 2-monoacylglycerols from algal oil and then synthesized TAGs containing caprylic acid (CA) mainly at the *sn*-1,3 position and DHA at the *sn*-2 position by reacting the monoacylglycerols with free CA in a solvent medium catalyzed by Lipozyme RM IM. In general, these multistage methods to obtain structured lipids result in higher yields of the desired TAGs, but their disadvantage is the requirement of further separation/purification steps such as evaporation and distillation, which might result in excessively high costs, considering industrial scale production processes.

A few research groups have also used DHASCO as a DHA source in the development of structured lipids for use as human milk fat analogues or as an ingredient in the production of infant formulas (*1*, *11*, *24*-*27*). This corroborates our preference for an SCO as a prime source of DHA, also considering that such a compound cannot be obtained from other sources and is crucial for the development and well-being of infants as well as adults (*2*).

Based on these considerations, the purpose of this study is to evaluate the influence of the lipase type and the amount ratio of FFAs in the substrate on an enzymatic acidolysis reaction to develop a structured lipid for the incorporation of CA, as a rapid source of energy, into the glycerol backbone of DHASCO.

## MATERIALS AND METHODS

### Materials

The single cell oil (CSO) rich in docosahexaenoic acid (DHA), DHASCO, was produced and kindly donated by Martek Biosciences Corporation (Columbia, MD, USA). Caprylic acid (98%) was obtained from Sigma-Aldrich, Merck (São Paulo, Brazil). Commercial immobilized lipases, namely Lipozyme TL IM, an *sn-*1,3 regiospecific lipase from *Thermomyces lanuginosus*, and Novozym 435 from *Candida antarctica,* were generously donated by Novozymes Latin America (Araucária, Brazil). All other reagents were of analytical or chromatographic grade (Merck, Darmstadt, Germany).

### Blend preparation

In order to evaluate the effect of molar ratio on the incorporation of caprylic acid (CA) by DHASCO, these compounds were blended in different molar proportions, namely 1:1 (BL1), 1:2 (BL2), 1:3 (BL3), 1:4 (BL4) and 1:5 (BL5). Blends were prepared by vigorous manual agitation in screw-capped glass bottles, stored under refrigeration, and protected from the incidence of light and contact with air.

### Batch acidolysis reactions

Blends (200 g) in the previously mentioned amount ratios were subjected to enzymatic acidolysis in a batch reactor consisting of a round-bottomed flask with precise temperature control during heating (C-MAG HS7; IKA, Königswinter, Germany) and a rod stirrer (RW 20; IKA) to maintain the agitation at a speed that keeps the lipase in suspension. Nitrogen was flushed into the flasks before the reactions started and then they were kept closed for 6 h. Lipozyme TL IM or Novozym 435 was added up to 10% by mass of substrate (*28*). Samples for the following analyses were identified according to Table 1.

**Table 1:** Symbols for different blends of single cell oil rich with docosahexaenoic acid (DHASCO) and caprylic acid (CA)

Sample	Lipase	*n*(CA)/*n*(DHASCO)
BL1	None	1:1
BL2	None	2:1
BL3	None	3:1
BL4	None	4:1
BL5	None	5:1
SL1-435	Novozym 435	1:1
SL2-435	Novozym 435	2:1
SL3-435	Novozym 435	3:1
SL4-435	Novozym 435	4:1
SL5-435	Novozym 435	5:1
SL1-TLIM	Lipozyme TL IM	1:1
SL2-TLIM	Lipozyme TL IM	2:1
SL3-TLIM	Lipozyme TL IM	3:1
SL4-TLIM	Lipozyme TL IM	4:1
SL5-TLIM	Lipozyme TL IM	5:1

### Purification of structured lipids

Due to the great amount of free fatty acids (FFA) present in some samples and partial acylglycerols in the structured lipids, samples were purified prior to TAG and FA composition analyses in order to isolate the TAG molecules. Lipid samples were diluted in *n*-hexane up to 200 mg/mL, passed through an aluminum oxide column (Merck) previously activated at 200 °C for 3 h, and then collected and evaporated with a nitrogen stream. The effectiveness of the purification process was evaluated with qualitative thin layer chromatography (TLC), where samples previously diluted in *n*-hexane were run through 0.25-mm silica gel plates (Merck) placed in an 80:20:1 (by volume) *n*-hexane/ether/acetic acid solvent system. Plates were revealed through exposure to iodine vapor.

### Free fatty acids

Free fatty acids (FFA) of DHASCO were determined by titration, using 0.1 M sodium hydroxide solution, according to AOCS official method Ca 5a-40 (*29*) and expressed as oleic acid equivalent (g/100 g).

### Fatty acid composition

DHASCO TAGs, their blends with FAs, and the purified structured lipids were converted into fatty acid methyl esters (FAMEs) according to Menezes *et al*. (*30*). Methyl esters for gas chromatographic analyses were prepared by saponifying 35 mg of samples with 0.5 mL of 0.5 mol/L methanolic sodium hydroxide solution. After the addition of this solution, samples were heated in hermetically sealed 20-mL flasks in a water bath at 90 °C for 10 min, cooled in an ice bath and then allowed to react with 1.5 mL of the esterifying solution. This esterifying solution had been previously prepared in a round-bottom flask attached to a condenser by refluxing for 15 min a blend of 2.0 g ammonium chloride, 60 mL methanol and 3.0 mL concentrated sulfuric acid, and finally stored in a hermetically sealed glass flask under refrigeration for a few weeks. Saponified samples with the esterifying solution were heated in a water bath at 90 °C for 10 min and then cooled in an ice bath.

FAME purification started with the addition of 5 mL of *n*-hexane and 10 mL of distilled water to the samples. Blends were vigorously shaken and, after some time at rest, a phase separation occurred. The upper organic phase containing the FAMEs was recovered with a pipette and washed thrice with 10 mL distilled water, while the bottom phase was properly discarded.

Analyses of FAMEs were carried out on a GC gas chromatograph, model 430 GC (Varian Chromatograph Systems, Walnut Creek, CA, USA), equipped with a CP 8412 auto injector. The Galaxie software (*31*) was used for quantification and identification of peaks. Injections were performed on a 100-m fused silica capillary column (internal diameter of 0.25 mm) coated with 0.2 μm of polyethylene glycol (SP-2560; Supelco, Bellefonte, PA, USA) using helium as the carrier gas at an isobaric pressure of 37 psi, linear velocity of 20 cm/s, makeup gas: helium at 29 mL/min at a split ratio of 1:50, injected volume 1.0 μL. The injector temperature was set to 250 °C and the detector temperature to 260 °C. The oven temperature was initially held at 60 °C for 7 min, programmed to increase to 135 °C at a rate of 15 °C/min and then held isothermally for 10 min. Then the temperature was programmed to increase again to 215 °C at a rate of 2 °C/min and then held isothermally for 40 min. Qualitative FA composition of samples was determined by comparing the retention times of the peaks produced after injecting the methylated samples with those of the respective standards of FAs. The composition was obtained by area normalization and expressed as mass fraction. All samples were analyzed in triplicate, and the reported values expressed as mean values.

### Triacylglycerol composition

Purified TAG samples were dissolved in acetone (5 mg/mL) and analyzed using a high-performance liquid chromatograph, model Prominence 20A (Shimadzu Corporation, Kyoto, Japan), equipped with an evaporative light scattering detector Shimadzu ELSD-LTII and two columns Supelcosil TM C18 (25 cm×4.6 mm×5 μm) (Supelco). Flow rate was initially set at 1 mL/min of a solvent mixture composed of acetone and acetonitrile (1:1), with an increasing linear gradient of chloroform of up to 20% within 60 min. This solvent composition was maintained for 20 min and finally returned to the starting composition for up to 80 min. Peaks were identified using pure TAG standards (Sigma-Aldrich, Merck, Darmstadt, Germany) and considering the order of elution according to the corresponding equivalent carbon number (*N*(C)_equivalent_), which is defined as the difference between the real number of carbon atoms in the aliphatic residues (CN) and twice the number of double bonds (N) per molecule. Two replicate analyses were performed and results expressed as mean values (*32*).

### Crystallization profiles

Thermal analysis was performed in a DSC 4000 calorimeter (Perkin-Elmer, Shelton, CT, USA) according to an adaptation of AOCS official method Cj 1-94 (*33*). Lipid samples weighing approx. 5 mg were placed into 50-μL aluminum pans (BO14-3017 container; PerkinElmer) and hermetically sealed (lid BO14-3003; PerkinElmer). After the pan with the sample had been placed in the oven, it was heated rapidly to 80 °C, held for 10 min to destroy crystal nuclei, then cooled down to -60 °C at a rate of 10 °C/min, and held at the isothermal holding temperature for 30 min. Samples were reused for further experiments. All experiments were conducted in triplicate, and the results expressed as mean values.

### Statistical analysis

Results of the determination of FA and TAG compositions were presented as average values with standard deviations, calculated with the software TIBCO Statistica v.12 (*34*) for Windows.

## RESULTS AND DISCUSSION

### DHASCO fatty acid composition

Free fatty acid (FFA) composition was analyzed to determine the quality of DHASCO, due to its high degree of unsaturation. The obtained results (0.15 g/100 g oleic acid equivalents) indicate that they could be appropriately used in the subsequent acidolysis reaction.

FFA composition of DHASCO is compared in Table 2 with the manufacturer's specifications (Martek Bioscience Corporation), with data previously reported by Hamam and Shahidi (*24*) and with tuna oil FA composition adapted from Hita *et al*. (*35*).

**Table 2:** Experimental free fatty acid (FFA) composition of single cell oil rich with docosahexaenoic acid (DHASCO) compared to the manufacturer’s specifications, a previously published DHASCO composition (*24*) and tuna oil (*34*)

Peak no.	FFA	*t*_R_/min	*w*(DHASCO)/%	Manufacturer’s specifications*	*w*(DHASCO)/%	*w*(tuna oil)/%
1	C10:0	22.0	0.6±0.0	max. 5.0	0.5±0.0	-
2	C12:0	24.0	4.3±0.1	max. 15.0	3.5±0.1	-
3	C14:0	26.9	12.0±0.1	max. 25.0	12.9±0.1	4.8
4	C16:0	30.4	11.0±0.2	max. 20.0	10.5±0.1	20.8
5	C16:1	31.6	2.2±0.0	max. 10.0	-	7.0
6	C18:0	34.3	0.7±0.1	max. 5.0	0.9±0.0	5.7
7	C18:1	35.1	22.1±0.3	max. 40.0	26.6±1.5	15.9
8	C18:2	36.6	1.0±0.1	max. 3.0	1.4±0.0	1.9
9	C22:6 *n*-3 DHA	47.3	45.6±0.5	approx. 40	37.1±0.4	20.1
10	Others	-	0.5	max. 5.0	6.6	23.8
	Total saturated		27.9		27.4	25.6
	Total unsaturated		71.6		66.0	50.6
Total			100.0		100.0	100.0

*t*_R_=retention time, *Martek Biosciences Corp.

DHASCO contains saturated and unsaturated FFAs within a wide chain length range, with different degrees of unsaturation with up to six double bonds. Medium-chain saturated FFAs account for 5.0 g/100 g of total FAs, among which capric and lauric acids are predominant, but most of them are long-chain FFAs, either saturated like myristic acid or highly unsaturated like DHA. The FFA present in the highest mass fraction is DHA (45.6 g/100 g), followed by oleic acid (22.1 g/100 g), while the mass fractions of the remaining FFAs are lower than 12 g/100 g. Since at least 71.6 g/100 g of DHASCO FFAs are unsaturated, this bulk oil is highly unsaturated. From the results shown in Table 2, where some unidentified peaks are grouped as ‘others’ totaling 0.5 g/100 g, it is correct to state that DHASCO FFA composition is in accordance with the manufacturer’s specifications.

Hamam and Shahidi (*24*), who also used DHASCO as a substrate for the production of structured lipids, reported a relatively similar FA composition, the main difference lying in the DHA content (37.1 g/100 g) that was 18.6% lower than that found in the present study. Oleic acid is the second most abundant FA in both studies, and the others are present in similar amounts. In addition, these authors do not mention palmitoleic acid content, but we found it at 2.2 g/100 g in this work. More recently, Teichert and Akoh (*36*) presented a DHASCO fatty acid profile where lauric, myristic, palmitic, oleic and docosahexaenoic acid molar fractions were 4.4, 9.4, 6.9, 27.8 and 44.0%, respectively. Pande *et al*. (*37*) have found a very similar composition, with the amount fractions 4.5 lauric, 10.3 myristic, 9.9 palmitic, 22.2 oleic and 44.1% docosahexaenoic acids as the major fatty acids. The compositions of DHASCO in these recently published papers are closer to what we found. Either those differences found by Hamam and Shahidi (*24*) could be simply due to common differences between batches or substantial improvements occurred along the last decade in the fermentation process to produce this single cell oil.

In most studies, DHASCO is presented as a more advantageous alternative to fish oil, which have lower DHA contents and undesirable flavor, and often contain environmental contaminants (*2*). Tuna oil appears to be an exception to most other fish oils, as it has one of the highest DHA content (Table 2) and a DHA to EPA ratio (4:1) very close to that of mother’s milk. Nonetheless, DHASCO contains more than double the DHA content of tuna oil.

### Triacylglycerol rearrangement

The TAG composition of DHASCO is presented in Table 3. The analytical method used to determine the TAG composition of the samples is not capable to distinguish between the regioisomers, therefore the TAG designated as ODD (with O and D meaning oleic acid and DHA, respectively), for example, can be the sum of ODD+DOD, in case they are both present. The main TAGs found in DHASCO are ODD (17.2%), MOD (12.9%), OOO (10.8%), DDD (8.7%), MPD (7.9%), PDD (6.6%) and OOD (5.3%) (see Table 3 for symbols), all other identified TAGs being present in amounts lower than 5.0% and the sum of unidentified peaks accounting for 9.8% of total TAGs. Despite the authors’ efforts, previously published results of DHASCO TAG composition were not found in the literature.

**Table 3:** Triacylglycerol (TAG) composition of single cell oil rich with docosahexaenoic acid

Peak no.	TAG	*N*(C)_equivalent_	*t*_R_/min	*w*(TAG)/%
7	DDD	30	14.188	8.7±0.2
8	LDD	32	15.239	4.1±0.2
9	MDD	34	16.562	2.3±0.1
10	ODD	36	17.798	17.2±0.1
12	PDD	36	19.414	6.6±0.0
13	LOD	38	20.348	2.7±0.1
14	MOD	40	20.953	12.9±0.1
16	MPD	40	22.803	7.9±0.2
19	OOD	42	25.882	5.3±0.1
20	POD	42	26.611	3.7±0.0
22	MMM	42	28.485	1.2±0.0
23	LOO	44	29.970	3.6±0.0
26	MOO	46	32.748	1.5±0.0
30	POO	48	37.380	1.2±0.0
32	OOO	48	39.554	10.8±0.8
35	MMD	52	45.706	0.6±0.0
	n.i.			9.8
	Total			100.0

L=lauric acid, M=myristic acid, P=palmitic acid, O=oleic acid, D=docosahexaenoic acid (DHA); n.i.=not identified, *t*_R_=retentiion time

**Fig. 1:**
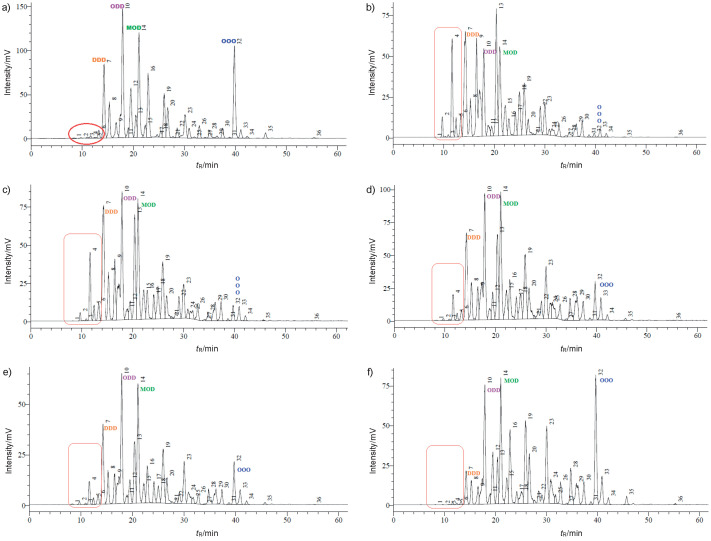
Triacylglycerol composition of single cell oil rich with docosahexaenoic acid (DHASCO) and of structured lipids obtained using Lipozyme TL IM as a catalyst at different *n*(caprylic acid)/*n*(DHASCO) in blends: a) DHASCO, b) SL1-TLIM (1:1), c) SL2-TLIM (2:1), d) SL3-TLIM (3:1), e) SL4-TLIM (4:1) and f) SL5-TLIM (5:1)

The primary TAGs possibly resulting from the acidolysis reaction between DHASCO and CA catalyzed by Lipozyme TL IM and Novozym 435 are CCD, CDD, CCM, CCP, CCO, CMD, CPD and COD (see Table 3 for symbols other than C, which stands for caprylic acid), while their respective equivalent carbon numbers (*N*(C)_equivalent_) are 26, 28, 30, 32, 32, 32, 34 and 34. TAGs should be eluted within retention times ranging from 10 to 16 min (Table 3), a region of the chromatogram characterized by numerous peaks very close to each other with coincident *N*(C)_equivalent_, but the absence of such chromatographic standards on the market prevented their quantification. Nonetheless, the general changes in DHASCO TAGs following acidolysis using Lipozyme TL IM as a catalyst can be observed in the chromatograms in Fig. 1.

The appearance of peaks before tridocosahexaenoyl glycerol (DDD) (*N*(C)_equivalent_=30) indicates the formation of TAGs with ECN<30, *i.e.* containing CA. While the sum of these peaks indicates the formation of not less than 8.4 and 5.3% of CA-containing TAGs in SL1-TLIM and SL2-TLIM samples, they accounted for only 2.4 and 2.0% in SL3-TLIM and SL4-TLIM, respectively, and were even absent from SL5-TLIM (see Table 1 for symbols). As the CA molar ratio was increased from 1 to 5, a decrease in CA incorporation by DHASCO TAG molecules took place.

**Fig. 2:**
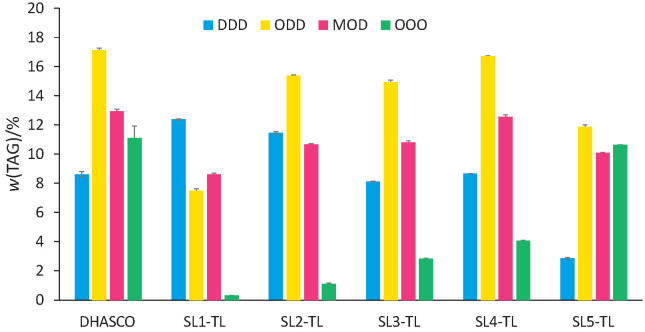
Main triacylglycerols (TAGs) present in single cell oil rich with docosahexaenoic acid (DHASCO) and in structured lipids synthesized using Lipozyme TL IM as a catalyst with different *n*(caprilic acid)/*n*(DHASCO) in blends: SL1-TL (1:1), SL2-TL (2:1), SL3-TL (3:1), SL4-TL (4:1) and SL5-TL (5:1). Abbreviations of fatty acids incorporated in TAGs, irrespective of their position on glycerol backbone: D=docosahexaenoic acid, O=oleic acid, M=myristic acid

Fig. 2 illustrates the mass fractions of the main TAGs of DHASCO and structured lipids obtained by acidolysis catalyzed by Lipozyme TL IM. The mass fraction of DDD, in general, progressively decreased from about 12.4 to 2.9% with increasing the CA/DHASCO amount ratio in the blend from 1:1 (SL1-TLIM) to 5:1 (SL5-TLIM), showing a redistribution of DHA among the structured TAGs, not only among the ones that incorporated CA, but also among the others. This can be inferred from the appearance of a great number of new peaks in the chromatogram region from 14 to 40 min (Fig. 1). These peaks, which correspond to TAGs that do not contain CA, suggest that transesterification among DHASCO TAGs may have occurred in preference to acidolysis with CA. Besides, the increasing heights of the pre-existing peaks in the same region corroborate the assumption of DHA redistribution among all TAGs. Concurrently, triolein (OOO) content in all structured lipids was, in general, lower than in DHASCO, thereby suggesting that this TAG was consumed in the reaction. On the other hand, the increasing amount of OOO, associated with a lower CA incorporation by DHASCO TAGs, as a function of an increased CA/DHASCO amount ratio in the blends, leads to the idea that CA reacted mainly with OOO, indicating a preference of Lipozyme TL IM for oleic acid. Aggelis *et al.* (*38*) studied the hydrolytic action of lipase from *Mucor miehei* for the preparation of polyunsaturated fatty acid methyl esters from borage and cod liver oil. This lipase preferentially hydrolyzed fatty acid esters with small number of double bonds and short aliphatic chains, probably due to the stereochemical hindrance of long aliphatic and polyunsaturated chains. Therefore, the fact that the oleic acid forming OOO TAGs can be hydrolyzed more quickly than other fatty acids such as DHA could explain the higher reduction of OOO instead of DDD or ODD during the incorporation of CA by DHASCO TAGs.

Hamam and Shahidi (*24*), who performed acidolysis of DHASCO and capric acid (C10:0) in blends with different amount ratios using lipase PS-30 from *Pseudomonas* sp., found that when the amount ratio of these substrates increased from 1:1 to 1:3, capric acid incorporation increased accordingly. In most cases, a high substrate molar ratio is actually able to shift the reaction equilibrium towards the product formation and to improve the acyl incorporation, but in some studies (*39*, *40*) the amount of FFAs incorporated into TAGs decreased at a molar ratio >3:1 in lipase-catalyzed acidolysis, owing to possible inhibition of lipase activity caused by excess fatty acids as a substrate (*41*).

**Fig. 3:**
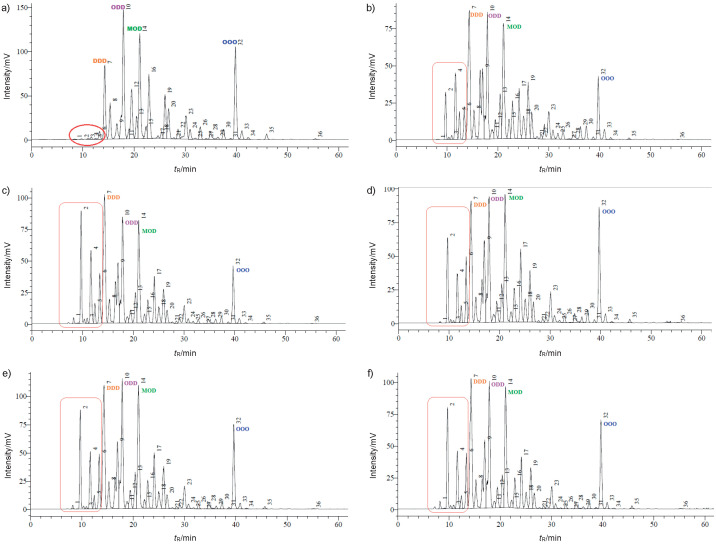
Triacylglycerol composition of single cell oil rich with docosahexaenoic acid (DHASCO) and of structured lipids obtained using Novozym 435 as a catalyst at different *n*(caprylic acid)/*n*(DHASCO) in blends: a) DHASCO, b) SL1-435 (1:1), c) SL2-435 (2:1), d) SL3-435 (3:1), e) SL4-435 (4:1) and f) SL5-435 (5:1)

A comparison between the chromatograms of DHASCO and structured lipids obtained by acidolysis catalyzed by Novozym 435 (Fig. 3) allows identifying two prominent emerging peaks with areas ranging from 3.1 to 8.6% and from 3.5 to 6.5%, respectively. Their retention times and *N*(C)_equivalent_ values <30, corresponding to DDD, suggest that they may consist of TAGs composed of two CAs and one DHA (CCD) with *N*(C)_equivalent_ of 26, or one CA and two DHAs (CDD) with *N*(C)_equivalent_ of 28. Although these peaks have the same retention times as those of structured lipids obtained using Lipozyme TL IM, their larger areas indicate a more efficient performance of Novozym 435 than with Lipozyme TL IM in CA incorporation by DHASCO. Moreover, structured lipids synthesized by Novozym 435 have a DDD content apparently 3 to 6% higher than DHASCO, which could be explained with the formation of CCM (*N*(C)_equivalent_=30), a TAG composed of two CAs and one myristic acid, having the same *N*(C)_equivalent_ as DDD and consequently leading to superposed peaks.

**Fig. 4:**
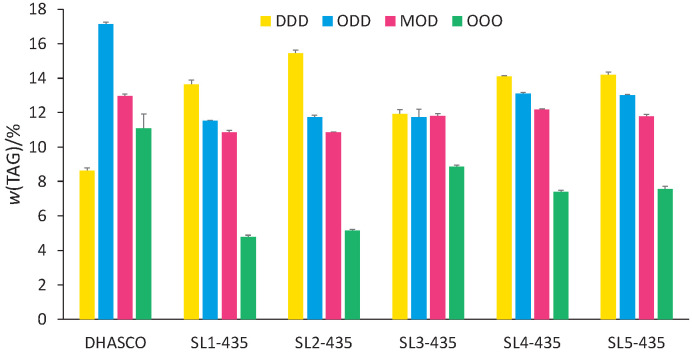
Main triacylglycerols (TAGs) present in single cell oil rich with docosahexaenoic acid (DHASCO) and in structured lipids synthesized using Novozym 435 as a catalyst with different *n*(caprilic acid)/*n*(DHASCO) in blends: SL1-435 (1:1), SL2-435 (2:1), SL3-435 (3:1), SL4-435 (4:1) and SL5-435 (5:1). Abbreviations of fatty acids incorporated in TAGs, irrespective of their position on glycerol backbone: D=docosahexaenoic acid, O=oleic acid, M=myristic acid

Triolein is present in higher mass fractions in structured lipids obtained with Novozym 435 than with Lipozyme TL IM, but in both systems its content was lower than in DHASCO (Fig. 4). ODD, MOD and OOO contents, in general, are lower than in DHASCO, suggesting that they were consumed in the acidolysis reaction, resulting in new TAGs. Taken as a whole, all Novozym 435-catalyzed SLs had similar TAG compositions, independently of CA/DHASCO molar ratio, which suggests that this parameter did not influence significantly CA incorporation by DHASCO. In contrast to what was inferred for Lipozyme TL IM-catalyzed structured lipids, *i.e.* redistribution of DHASCO FFAs among its own TAGs, the peaks of these new TAGs obtained with Novozym 435 are concentrated in the chromatogram region corresponding to retention times lower than 14 min, with *N*(C)_equivalent_ typical of CA-containing TAGs.

Ma *et al.* (*42*) were able to achieve flexible concentrations of DHA and EPA in glycerides simultaneously with biodiesel production *via* a two-step process catalyzed by lipases. In the first step, Novozym ET2.0 acted selectively for the concentration of DHA and EPA *via* ethanolysis of fish oil. Then, the oil phase was subjected to molecular distillation, when a biodiesel could be obtained. The heavy phase was transesterified with DHA- or EPA-rich ethyl ester catalyzed by immobilized lipase Novozym 435 for further flexible enrichment of DHA and EPA. The same research group had achieved similar results in a previous publication using low-grade fish oil as substrate and Novozyme NS81006 and 435 as catalysts (*43*). Higher production yields of glycerides enriched with *n*-3 can be achieved with processes consisting of two or more steps with infinite possibilities of synthesis and separation combinations. Obviously, these sophisticated methods are very challenging and costly, thereby not accessible to any industrial plants.

The operational stability of immobilized lipases such as Novozym 435 can be improved when structured lipid synthesis is performed in solvent reaction media. Squalene could act as a solvent without being vaporized from the reaction system under vacuum during the enzymatic synthesis of ether lipids rich in DHA *via* transesterification of alkylglycerols obtained from shark liver oil and DHA-enriched ethyl esters catalyzed by Novozym 435 (*44*). In another case, triglycerides and ethyl esters containing *n*-3 fatty acids from fish oil were employed as the substrates for transesterification catalyzed by immobilized lipase using imidazolium-based ionic liquid systems. The total EPA and DHA content in the resulting TAG was 11.74% higher than that of the TAG produced in a solvent-free reaction system, showing that the addition of solvents to the reaction medium can favor the production of *n*-3-enriched TAGs using immobilized lipases (*45*).

### Thermal behavior

**Fig. 5:**
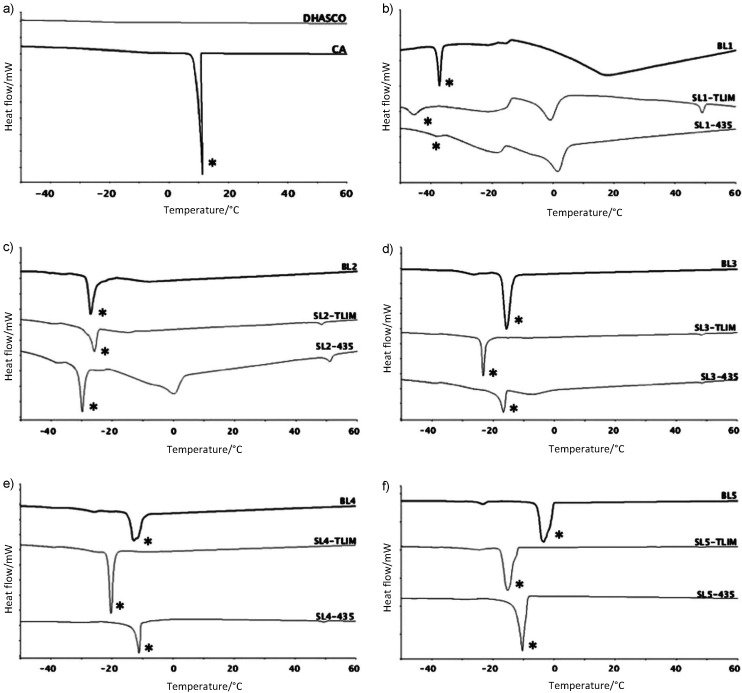
Crystallization curves of raw materials, their blends and respective structured lipids, showing the heat flow as a function of temperature (°C): a) single cell oil rich with docosahexaenoic acid (DHASCO) and caprylic acid blend, and blends with different *n*(caprilic acid)/*n*(DHASCO): b) BL1, SL1-TLIM and SL1-435, c) BL2, SL2-TLIM and SL2-435, d) BL3, SL3-TLIM and SL3-435, e) BL4, SL4-TLIM and SL4-435, and f) BL5, SL5-TLIM and SL5-435. Abbreviations are the same as in Table 1. Symbols for different blends of single cell oil rich with docosahexaenoic acid (DHASCO) and caprylic acid (CA). *Peaks generated by caprylic acid crystallization

Oils and fats are complex molecular systems that mainly consist of different TAGs, some diacylglycerols and monoacylglycerols, and free fatty acids. Thus, melting and crystallization of oil do not occur at a single temperature, but within a wide range (*46*). Fig. 5 illustrates the crystallization curves of raw materials, blends and structured lipids obtained by differential scanning calorimetry (DSC).

DHASCO did not crystallize during cooling down to -60 °C, indicating especially difficult packing of its very long and highly unsaturated molecules. Caprylic acid led to a sharp peak at 11.3 °C. Similar sharp peaks persisted in all blends and structured lipids, as they were not purified to eliminate the excess CA added to DHASCO in order to perform the acidolysis reaction, all marked with an asterisk in the figure. Interestingly, these sharp peaks caused by CA’s saturated short chain crystallization were delayed in the presence of DHASCO. As the CA content in the blend increased, the influence of long polyunsaturated DHASCO TAGs was reduced, and the crystallization temperature of the blend became closer to that of pure CA.

The blend containing DHASCO and AC in 1:1 molar ratio (BL1) had only one sharp crystallization peak at -39.7 °C, corresponding to CA, a profile significantly different from those of the structured lipids obtained with Lipozyme TL IM (three new peaks at 48.7, -0.7 and -17.5 °C) and Novozym 435 (two new peaks at 1.6 and -18.5 °C). The appearance of additional crystallization peaks using Lipozyme TL IM (48.6 °C) and Novozym 435 (51.1 and 0.0 °C) in structured lipids synthesized from BL2, which showed a single peak at -27.3 °C, is worth mentioning. These results taken together suggest not only the actual occurrence of interesterification reaction, but also the formation of new CA-containing TAGs, as previously suggested by the new TAG compositions of these SLs.

For BL3, significant changes took place in the crystallization profiles of structured lipids synthesized with the two enzymes, both between Lipozyme TL IM and Novozym 435 themselves and compared to what had been observed before the interesterification (BLs). Peaks with larger areas occurred at lower temperatures in structured lipids, especially the one obtained with Lipozyme 435 (-23.3 °C) than in the blend (-15.8 °C). Although there was no formation of new peaks with significant areas in SLs obtained from BL4 and BL5, changes can be observed in the crystallization temperature, suggesting a rearrangement of DHASCO fatty acids among its own TAGs rather than CA incorporation resulting from acidolysis. SL5-435 and SL5-TL IM had their most significant peaks at lower temperatures (-10.3 and -14.7 °C, respectively) than that before the interesterification (-4.1 °C).

Pina-Rodriguez and Akoh (*25*) synthesized SLs from amaranth oil by adding palmitic acid to the *sn-2* position in the TAGs and then incorporating DHA mainly at the *sn*-1,3 positions using Lipozyme RM IM. The thermograms obtained for their structured lipids showed a wider melting range due to the presence of small portions of TAG with higher melting points, according to the authors, as its palmitic acid content was higher and that of linoleic acid lower than in amaranth oil.

Therefore, we can conclude that new TAG molecules appeared in structured lipids, leading to the formation of new crystallization peaks or changes of the peaks shown by the initial blends. This allowed proving, through the comparative analysis of thermal behavior of structured lipids obtained with Novozym 435 and Lipozyme TL IM, the occurrence of both transesterification and acidolysis reactions, depending on the CA proportion in the substrate. Finally, corroborating the TAG composition results presented previously, lower concentrations of excess CA in the substrate led to harsher transformations in the original DHASCO TAGs, either using Lipozyme TL IM or Novozym 435, resulting in lipids with differentiated thermal profiles because of new TAGs formation.

## CONCLUSIONS

Although fish oil has long been considered the main source of ω-3 fatty acids, it has more recently been replaced by single cell oil due to the common presence of environmental contaminants and the perception of undesirable flavor by the consumer. These types of oil derived from microorganisms, besides representing an alternative and sustainable source of docosahexaenoic acid (DHA), are considered to contribute to the DHA uptake metabolism and its incorporation into the brain and retinal lipids. The results obtained herein reveal that enzymatic acidolysis can be a successful method to incorporate caprylic acid (CA) into the triacylglycerols (TAGs) in the single cell oil rich with DHA, producing structured lipids capable of providing energy as well as DHA in a more absorbable manner. As the *n*(CA)/*n*(DHA) from single cell oil (SCO) in the substrate was increased from 1:1 to 5:1, a decrease in the formation of CA-containing TAGs took place. Additionally, the regiospecific lipase Lipozyme TL IM seemed to induce transesterification among DHASCO TAGs preferably for acidolysis with CA, which was directly proportional to the increase of CA ratio in the substrate. Also, when CA was incorporated, a decrease in the content of triolein was observed, indicating a preference of Lipozyme TL IM for oleic acid. In reactions catalyzed by the non-regiospecific lipase Novozym 435, a higher incorporation of CA by DHASCO TAGs was observed than with Lipozyme TL IM, independently of the *n*(CA)/*n*(DHASCO), which suggests that this parameter did not influence significantly CA incorporation. The thermal analysis also suggested the appearance of new TAG molecules in structured lipids, which led to the formation of new crystallization peaks or changes in the peaks observed in the initial blends. A comparative analysis of the crystallization behavior of the structured lipids obtained with Novozym 435 and Lipozyme TL IM supports the hypothesis of the occurrence of both transesterification and acidolysis reactions, revealing that lower concentrations of excess CA in the substrate led to harsher transformations in the original DHASCO thermograms. These results taken as a whole revealed the importance of combining the choice of the type of lipase, either regiospecific or not, with the proportions of excess free fatty acids in acidolysis reactions when aiming to produce structured lipids as a source of DHA.
